# Protective Effects of Melatonin on Neurogenesis Impairment in Neurological Disorders and Its Relevant Molecular Mechanisms

**DOI:** 10.3390/ijms21165645

**Published:** 2020-08-06

**Authors:** Joseph Wai-Hin Leung, Kwok-Kuen Cheung, Shirley Pui-Ching Ngai, Hector Wing-Hong Tsang, Benson Wui-Man Lau

**Affiliations:** 1 Department of Biology, University of Ottawa, Ottawa, ON K1N 6N5, Canada; wleung2@uottawa.ca; 2 Regenerative Medicine Program, Ottawa Hospital Research Institute, Ottawa, ON K1H 8L6, Canada; 3 Department of Rehabilitation Sciences, The Hong Kong Polytechnic University, Hong Kong, China; alexkk.cheung@polyu.edu.hk (K.-K.C.); shirley.ngai@polyu.edu.hk (S.P.-C.N.)

**Keywords:** melatonin, neurogenesis, neural stem cell, neurogenesis impairment, neurological disorder, molecular mechanism, review

## Abstract

Neurogenesis is the process by which functional new neurons are generated from the neural stem cells (NSCs) or neural progenitor cells (NPCs). Increasing lines of evidence show that neurogenesis impairment is involved in different neurological illnesses, including mood disorders, neurogenerative diseases, and central nervous system (CNS) injuries. Since reversing neurogenesis impairment was found to improve neurological outcomes in the pathological conditions, it is speculated that modulating neurogenesis is a potential therapeutic strategy for neurological diseases. Among different modulators of neurogenesis, melatonin is a particularly interesting one. In traditional understanding, melatonin controls the circadian rhythm and sleep–wake cycle, although it is not directly involved in the proliferation and survival of neurons. In the last decade, it was reported that melatonin plays an important role in the regulation of neurogenesis, and thus it may be a potential treatment for neurogenesis-related disorders. The present review aims to summarize and discuss the recent findings regarding the protective effects of melatonin on the neurogenesis impairment in different neurological conditions. We also address the molecular mechanisms involved in the actions of melatonin in neurogenesis modulation.

## 1. Introduction

Adult neurogenesis, namely, generation of new neurons, was discovered in the 1960s [1], while its involvement in behaviors and diseases were discovered in the last two decades since the discovery of the neurogenesis-promoting property of antidepressants [2]. Neurogenesis is implicated in different behaviors and brain functions [3]. For instance, neurogenesis in the subventricular zone (SVZ) is hypothesized to be responsible for the sexual and olfactory behavior, whereas neurogenesis taking place in the hippocampus is important for the learning and memory processes as well as pattern separation [4,5,6,7].

Melatonin is a hormone secreted by the pineal gland in the mammalian brain. Its first identified role is to regulate the circadian rhythm and the sleep–wake cycle [8]. Previously, it was hypothesized that melatonin is synthesized in the cytosol of cells; however, recent findings have shown that mitochondria is the original site of melatonin synthesis [9]. Melatonin synthesis has been found in mitochondria of different cells including oocytes, pinealocytes, endothelial cells, plant cells, and neurons [10]. Serotonin *N*-acetyltransferase (SNAT) is a melatonin synthetic enzyme, whereas *N*-acetyl-coenzyme A is an irreplaceable substrate of SNAT. Since *N*-acetyl-coenzyme A is mainly synthesized in mitochondria instead of cytosol, this may further support the idea that melatonin is primary produced in mitochondria but not cytosol [10]. It was suggested that the main function of melatonin in mitochondria is to protect mitochondria from oxidative stress [11]. Melatonin activates two high affinity receptors—MT1 (Mel1a, MTNR1A) and MT2 (Mel1b, MTNR1B) [8]. MT1 and MT2 receptors are G protein-coupled receptors that are encoded by genes on human chromosome 4 and chromosome 11 [12]. Considering the functions of MT1 and MT2 receptors, apart from regulating the circadian rhythm, they are also responsible for different physiological functions, including reproduction, cardiovascular regulation, and immune function [13,14,15,16]. Melatonin is neuroprotective against the central nervous system (CNS) disorders, particularly the neurodegenerative diseases such as Parkinson’s and Alzheimer’s disease [17,18]. In addition, it also protects the brain from ischemia injury. The neuroprotective effects of melatonin are mainly attributed to its antioxidant, anti-inflammatory, and anti-apoptotic properties, wherein the melatonin receptors are involved [19]. It has been claimed that the mitochondrial MT1 but not the plasma membrane MT1 is responsible for the protective effects of melatonin on the cerebral damage caused by ischemia, but this requires further confirmation [20].

Until recently, the neurogenic property of melatonin was undiscovered, with melatonin being found to play an important role in the regulation of neurogenesis [19]. Since neurogenesis impairment is linked to different CNS disorders, it is worth studying the effects of melatonin on neurogenesis impairment as well as the respective molecular mechanisms in neuropathological conditions. Here, we summarize and discuss the most updated findings in terms of this aspect.

## 2. Roles of Melatonin in Modulation of Neurogenesis

**Figure 1:**
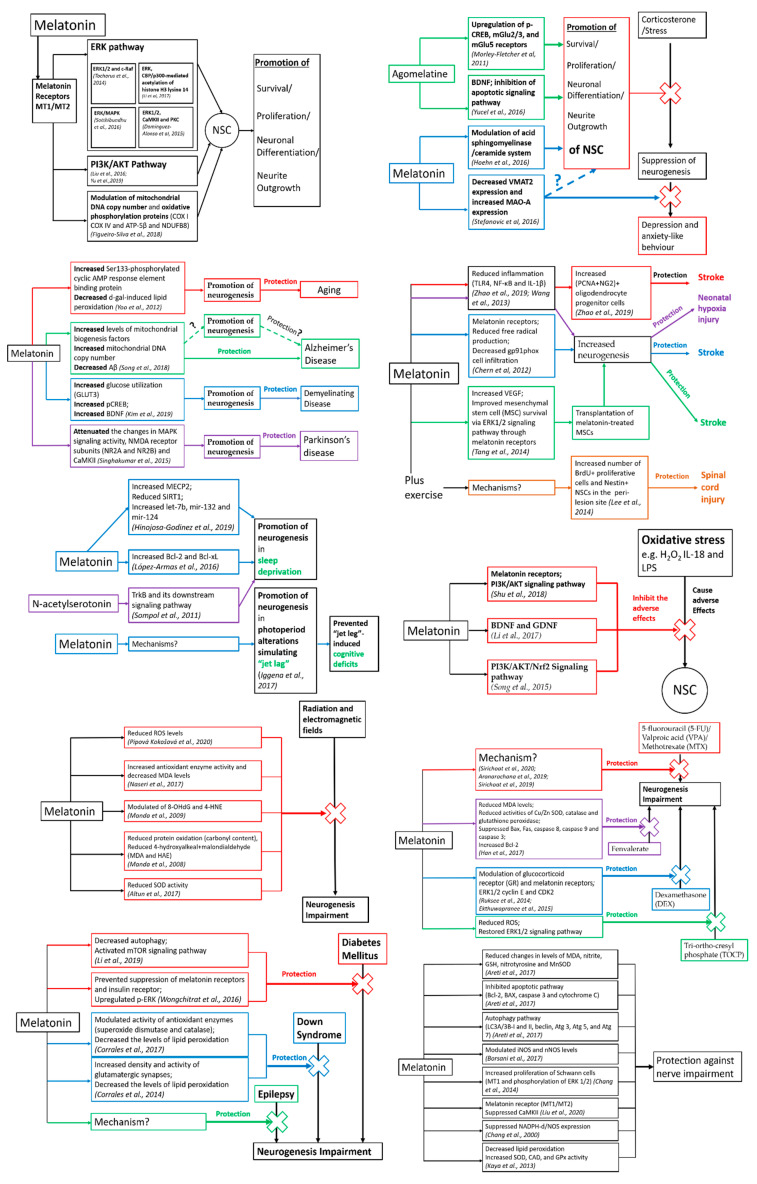
Effects of melatonin and its receptor agonist/precursor on neurogenesis and nerve regeneration and the respective molecular mechanisms in different neuropathological conditions.

Melatonin has been shown to be involved in the modulation of neurogenesis in both in vivo and in vitro models [21] (Figure 1).

### 2.1. In Vitro Model

Melatonin promotes viability, proliferation, and neuronal differentiation of mouse embryonic cortical neural stem cells (NSCs) [22,23,24]. It enhances differentiation of embryonic NSCs into neurons through melatonin receptors (MT1/MT2) with the CBP/p300-mediated acetylation of histone H3 lysine 14 via extracellular signal-regulated kinase (ERK) signaling pathway [23]. Similar effects on the neural stem cell line were observed, where the expression of neuronal markers increased in the PC12 and C17.2 NSCs after treatment of melatonin via the activation of the PI3K/Akt pathway [25,26]. With the application of melatonin receptor antagonist luzindole, the effects of melatonin on the PC12 cells was abolished, leading to reduction in neurite outgrowth and decrease in the number of mature neurons [25]. In another cellular model, melatonin enhanced dopaminergic neuronal differentiation of embryonic day 14 (E14) rat midbrain NSCs, with the effect being potentially brought out by the increased production of brain-derived neurotropic factor (BDNF) and glial cell line-derived neurotrophic factor (GDNF) in the NSCs [27]. Timing of melatonin treatment was found to be critical in the differentiation of NSCs. It was shown that melatonin promoted 1% fetal bovine serum (FBS)-induced neuronal differentiation during the proliferation period of E15.5 ganglionic eminence NSCs but it decreased neuronal differentiation of the NSCs during the differentiation period [28]. Melatonin also promoted the neuronal differentiation of amniotic fluid mesenchymal stem cells and mouse-induced pluripotent stem cells, in which ERK/CaMKII and PI3K/Akt signaling pathways were, respectively, involved [29,30]. In adult rat hippocampal organotypic culture experiments, melatonin was found to promote dendritogenesis by the activation of CaMKII, protein kinase C (PKC), and ERK1/2 with partial involvement of the melatonin receptors [31,32]. These results support the roles of melatonin on neurogenesis (Table 1).

### 2.2. In Vivo Models

Melatonin promotes adult neurogenesis in different models. From gain-of-function experiments, when mice were administered 8 mg/kg body weight (BW) melatonin daily for 7 or 14 days exogenously, the number of doublecortin-positive (DCX+) neuronal precursor cells in the dentate gyrus (DG) increased [38]. In addition, melatonin increased the structural plasticity of mossy fiber projection in the 8-week-old mice [39]. The roles of melatonin on adult neurogenesis were further confirmed by the loss-of-function pinealectomy experiments. In the pinealectomy experiments, the pineal gland, which is the primary production site of melatonin, was surgically removed from the animals. After rats were pinealectomized, the levels of melatonin decreased and neurogenesis in the hippocampus declined; however, the neurogenesis impairment was reversed by the melatonin treatment [40]. Male offspring of the mouse mothers, which were pinealectomized before pregnancy, also exhibited neurogenesis disruption, but the impairment could be alleviated by the melatonin treatment [41]. Apart from promoting neurogenesis, melatonin enhanced survival and dendritic maturation of the immature neurons in the hippocampus [33] (Table 2).

The neurogenic effects of melatonin were observed when the mice were treated at a dose as low as 2 mg/kg BW daily for 7 days [24]. This dosage of melatonin increased the number of BrdU+/NeuN+ cells in the DG and modulated the mitochondrial DNA copy number and oxidative phosphorylation proteins, including COX I, COX IV, ATP-5β, and NDUFB8 in the hippocampus [24]. In the aging animal studies, melatonin delayed the decline of the hippocampal neurogenesis in the 6- to 9-month-old aging mice [42]. Increased cell proliferation and higher number of calretinin+ neurons were observed in the DG in the aging mice which received the melatonin treatment [42,43]. Moreover, melatonin potentiated the running-wheel activity-induced cell survival and neurogenesis in the DG in 2–3-month-old mice [44] (Table 2).

Metalation receptors are involved in adult neurogenesis. When mice were subjected to 10 mg/kg luzindole daily for 14 days, DCX+ neuronal precursor cells and Ki-67+ proliferative cells in the subgranular zone (SGZ) reduced [45]. Ex vivo studies also showed that melatonin increased survival, proliferation, and differentiation of adult SVZ, hippocampal, and spinal cord NSCs through the melatonin receptors via the ERK/MAPK and PI3K/Akt signaling pathways [33,34,35,36,37] (Table 1 and Table 2).

## 3. Melatonin and Depression

According to the neurogenesis hypothesis of depression, the disturbance of mood and emotion is linked to the impairment of neurogenesis, and restoration of neurogenesis would be a critical factor for the remission from the disease [2,46,47,48]. If neurogenesis is suppressed, the beneficial effects of antidepressants would be abolished, which may indicate that reversing neurogenesis deficits is a potential therapeutic target of depression [49]. The protective effects of melatonin on depression has been well documented. Depression-like behavior in male BALB/c mice was reduced when they were treated with 5 and 10 mg/kg BW melatonin daily for 7 days [50]. Melatonin reduced the immobility behavior of the mice in the forced swim test [50], and coincidently, neurogenesis and dendrite maturation in those mice were promoted [50]. Corticosterone (CORT) contributes to the onset of depression-like behavior in rodents. The CORT depression model is a well-established model for studying depression. It was shown that 3-week daily treatment of 10 mg/kg BW melatonin prevented CORT-induced reduction in cell proliferation in the DG and reduced depression- and anxiety-like behavior of the mice in the forced swim test, open field test, and novelty suppressed feeding test [51]. The anti-depressive and neurogenetic effects of melatonin was potentially brought about by the inhibition of the acid sphingomyelinase/ceramide system as well as the decrease in vesicular monoamine transporter 2 (VMAT2) levels and increase in the monoamine oxidase A MAO-A levels in the hippocampus [52,53]. Using combination treatment of melatonin and citalopram (MLTCITAL), 14-day daily treatment of 2.57 mg/kg BW MLTCITAL promoted neurogenesis and reduced depression-like behavior in adult mice [54] (Table 3).

Agomelatine is a mixed MT1/MT2 melatonin receptor agonist and 5HT2C serotonin receptor antagonist. Agomelatine was able to reverse the decline of neurogenesis regulators including p-CREB, mGlu2/3, and mGlu5 receptor levels in the hippocampus, and prevented the neurogenesis impairment and depression- and anxiety-like behaviors in the adult male offspring of mouse mothers who were subjected to restraint stress during pregnancy [55]. In addition, agomelatine also protected against light stress-induced neurogenesis deficits by upregulating BDNF levels and inhibiting the apoptotic signaling pathway [56]. It was also reported that agomelatine increased neurogenesis via the 5HT2C receptor with the activation ERK1/2, protein kinase B, and GSK3β [57,58] (Table 3).

## 4. Melatonin, Aging, and Neurodegenerative Diseases

Melatonin can decelerate the progress of aging. Melatonin improved spatial memory deficits in the mouse model of d-galactose (d-gal)-induced aging and restored the reduction of Ki67+ proliferative cells and DCX+ neuronal precursor cells caused by the d-gal in the DG [59]. Senescence-accelerated (SAMP8) mice is another mouse model of aging, which presented age-related defects such as cognitive disability and motor dysfunction [60]. When the SAMP8 mice were given melatonin treatment from the ages of 1 month to 10 months old, the levels of acetylated p53, NF-κB, and amyloid β (Aβ) in the brain decreased and the levels of α-secretase and Bcl-2_XL_ increased, which suggested that melatonin exerted anti-aging effects by modulating the pro-survival and pro-death signals in the brain [61]. However, the study did not examine the relationship between the survival/death signals and neurogenesis (Table 4).

Parkinson’s disease (PD) is a neurodegenerative disease that is linked to motor and learning dysfunction [67]. Melatonin attenuates neurogenesis impairment in different PD animal models. For instance, 7-day treatment of 5 mg/kg BW melatonin reversed the decline of Nestin, DCX, and Beta-III tubulin expression in the hippocampus in the methamphetamine (METH)-induced PD mice by modulating the MAPK signaling activity, N-methyl-d-aspartate (NMDA) receptor subunits (NR2A and NR2B), as well as CaMKII levels [62]. Transplantation of 25 μM melatonin-pretreated SVZ NSCs to the hippocampus and striatum also improved neuronal restoration in the 1-methyl-4-phenyl-1,2,3,6-tetrahydropyridine (MPTP)-induced PD mice [63]. Moreover, combination treatment of melatonin and NSC transplantation rescued tyrosine hydroxylase (TH) neurons and attenuated the behavioral deficits in the 6-hydroxydopamine (6 OHDA)-induced PD mouse model [64]. In the in vitro experiments, melatonin was shown to prevent the METH-induced inhibition of proliferation of NSCs by modulating the tumor suppressor p53 and cycle inhibitor p21CIP as well as regulating the CaMKII, NR2A, and NR2B [68] (Table 4 and Table 5).

Apart from Parkinson’s disease, melatonin was found to rescue neurogenesis impairment in demyelinating disease. Melatonin prevented reduction of DCX+ neuronal precursor cells and Ki-67 proliferating cells in the DG through the phosphorylation of CREB and upregulation of glucose transporter 3 (GLUT3) and BDNF levels [65]. Melatonin is also neuroprotective against Alzheimer’s disease (AD) [63,66]. Melatonin alleviated the AD-induced spatial learning and memory deficits by reducing the Aβ levels and modulating the mitochondrial biogenesis factors and DNA copy number in the brain [66]. However, the role of melatonin in the regulation of neurogenesis in AD has not yet been fully studied (Table 4).

## 5. Melatonin and Central Nervous System (CNS) Injury

Melatonin was shown to confer neuroprotective effects in stroke with yet elusive mechanisms. It was hypothesized that melatonin protected against stroke due to its anti-inflammatory and anti-oxidant properties. Recent studies reported that melatonin may also promote stroke recovery by regulating post-stroke neurogenesis. When mice were subjected to the middle cerebral ischemic/reperfusional injury (CI/R), it was found that melatonin treatment could reduce the post-stroke free radical production, increasing the number of the DCX+ neuroblasts and Ki67+ proliferative cells in the peri-infarcted area (e.g., the cortex) [69]. For those Ki-67+ cells, they were not only DCX+, but the majority of them were also Nestin+ and NG2+ [69]. Nestin and NG2 are the markers of NSC and oligodendrocyte precursors, respectively. The findings were further supported by another study that showed that melatonin increased PCNA+ and NG2+ oligodendrocyte progenitor cells in the SVZ and white matter in the rat after the focal cerebral ischemia with the downregulation of inflammatory factors, including TLR4, NF-κB, and IL-1β [70] (Table 6).

On neurological functions, melatonin improved motor and coordination of post-stroke mice in the grip strength and rotarod tests [71]. Melatonin also attenuated the hyperactivity and anxiety behavior of the mice in the open field test after stroke [71]. It was found that the improvements were associated with the promotion of endogenous neurogenesis in the lateral ventricle, striatum, and cortex after melatonin treatment [71]. Nevertheless, in the global forebrain ischemia experiment, which used 3- to 4-month-old Mongolian gerbils as the animal model, it was found that chronic but not acute melatonin treatment could improve the post-stroke behavioral outcome [72], and instead of enhancing the post-stroke neurogenesis, both chronic and acute melatonin suppressed the increased neurogenesis after stroke [72] (Table 6).

Melatonin facilitated the efficiency of post-stroke mesenchymal stem cell (MSC) transplantation therapy. Melatonin enhanced the survival of MSCs through the ERK1/2 signaling pathway via the melatonin receptors [73]. When the MSCs were pretreated with 5 mM melatonin for 24 h and transplanted to the ipsilateral striatum in the rats after stroke, the rats exhibited improved behavioral outcomes with an increase in the levels of neurogenesis and angiogenesis. The effects may be brought by the upregulation of vascular endothelial growth factor (VEGF) after the transplantation therapy [73] (Table 6).

Apart from stroke, melatonin protects against spinal cord injury (SCI). Melatonin treatment increased dendritic spine density in the peri-lesion site in the rats after SCI [74]. When the SCl rats received combined treatment of melatonin and treadmill exercise training, the hindlimb function in the injured animals were significantly improved in the Basso, Beattie, and Bresnahan (BBB) locomotor recovery scale. Meanwhile, the combination treatment also increased the number of BrdU+ proliferative cells and NSCs in the peri-lesion area [74] (Table 6).

In addition to SCI, melatonin was found to be protective on early-life CNS injuries such as perinatal hypoxia. Melatonin prevented microglial activation and downregulated the inflammatory mediators (e.g., TNFα, IL-1β, and nitric oxide) in the PND1 mice, which were subjected to the hypoxia at 5% oxygen and 95% nitrogen for 2 h [75]. Moreover, melatonin promoted neurogenesis and improved long-term deficits of the hypoxia-injured mice. It also attenuated the sensorimotor and locomotor function impairments, learning and memory deficits, and hyperactivity behavior of the mice 30 days after hypoxia [75]. In the in vitro study, when E12.5 mouse cortical NSCs were treated with 100 nM melatonin before being subjected to 12-h 95% *N*_2_ and 5% CO_2_ hypoxia incubation, the proliferation and neuronal differentiation of NSCs were restored by the phosphorylation of ERK1/2 via the MT1 receptor [77] (Table 6 and Table 7).

miR-363 was upregulated in the vitamin A deficiency (VAD)-induced congenital spinal deformities model. It was found that melatonin could promote proliferation, increased Nestin expression, and enhanced neuronal differentiation of the miR-363-transfected E13.5 rat NSCs [78]. In another CNS injury model, rats were exposed to kainic acid by intracerebroventricular administration on PND7 for the induction of neurodevelopmental injury. Melatonin protected against kainic acid-induced neurodevelopmental injury by preventing hippocampal neuronal loss without altering neurogenesis [76] (Table 6 and Table 7).

## 6. Melatonin and Sleep Deprivation

Sleep deprivation (SD) suppresses neurogenesis in the adult hippocampus [79]. SD also causes adverse effects on neurological behavioral outcomes [80]. It is speculated that hippocampal neurogenesis impairment is responsible for SD-induced behavioral deficits (Table 8).

However, melatonin could rescue the neurogenesis deficits in SD. When mice were subjected to SD for 96 h, a 4-day daily treatment of 10 mg/kg BW melatonin restored the SD-induced reduction in the number of Sox2+/BrdU+ NSCs in the DG by increasing the levels of methyl-CpG-binding protein 2 (MECP2) and decreasing the levels of Sirtuin 1 (SIRT1) [81]. In addition, melatonin could also increase the number of BrdU/Nestin+ NSCs in the SGZ in SD mice by upregulating the Bcl-2 and Bcl-xL levels [82]. *N*-acetylserotonin (NAS) is an immediate precursor of melatonin. It was found that NAS could prevent the suppression of NSC proliferation induced by SD via the TrkB signaling pathway [83] (Table 8).

Apart from SD, melatonin protected against the photoperiod alterations simulating “jet lag” [84]. Melatonin prevented the reduction of cell proliferation and attenuated the cognitive deficits caused by “jet lag” [84]. However, the underlying mechanism remains unclear (Table 8).

## 7. Melatonin, Inflammation, and Oxidative Stress

Due to its antioxidant properties, melatonin helps reduce inflammation and oxidative stress. Melatonin can scavenge free radicals, reactive oxygen species (ROS), and reactive nitrogen species (RNS) directly [85]. It can also scavenge hydrogen peroxide (H_2_O_2_) and neutralize the toxic hydroxyl radicals [86]. Inducible NO synthase (iNOS) generates nitric oxide (NO), which is a free radical [87]. Excessive amount of NO generated by iNOS can cause cytotoxic changes in cells [87]. Melatonin is able to inhibit iNOS and decrease NO levels [88,89]. It was also reported that, by regulating NOS expression, melatonin protected the brain in terms of pathological conditions such as ischemic brain injury [90]. In addition, pretreatment of melatonin was found to reduce nitric oxide (NO) levels and prevent E14 cortical NSCs from apoptosis after lipopolysaccharide (LPS) exposure [91] (Table 9).

Melatonin protects NSCs against inflammation and oxidative stress, not only through the regulation of NO levels. The study also showed that melatonin protected NSCs from LPS-induced cell death by activating the PI3K/Akt/Nrf2 signaling pathway [91] (Table 9).

H_2_O_2_ induces oxidative stress to iPSC-derived NSCs. H_2_O_2_ was shown to decrease proliferation and viability of iPSC-derived NSCs and reduced their mitochondrial membrane potential [92]. Melatonin, however, could reverse all the adverse effects induced by H_2_O_2_ by the activation of the PI3K/Akt signaling pathway via the melatonin receptors [92]. IL-18 is a cytokine that is detrimental to the NSCs. Melatonin, moreover, could promote the production of BDNF and GDNF in the IL-18-stimulated NSCs and suppressed the IL18-induced inhibition of proliferation, neurosphere formation, and neuronal differentiation of the NSCs [93] (Table 9).

## 8. Melatonin and Neurogenesis Impairment Caused by Environmental Factors

Neurogenesis impairment can be caused by environmental factors, which leads to different neurological deficits. Scopolamine is a prescription drug used for the prevention of nausea and vomiting. When mice were treated with scopolamine for 2 weeks, it was found that the number of DCX+ neuronal precursor cells and Ki67+ proliferative cells decreased in the DG, wherein the mice exhibited spatial learning and short-term memory impairments in the Morris water maze test and passive avoidance test [94]. However, when the mice were treated with melatonin, neurogenesis was restored and behavioral deficits were attenuated [94]. Apart from scopolamine, other drugs and chemicals such as 5-fluorouracil (5-FU), valproic acid (VPA), and methotrexate (MTX) also caused hippocampal neurogenesis impairment in rats, but the impairments could be reversed by melatonin treatment [95,96,97] (Table 10).

Dexamethasone (DEX) is a type of high potency corticosteroid medication. It causes depressive-like behavior and neurogenesis disruption in mice after exposure. It was found that pre-treatment of melatonin could prevent DEX-induced abnormality by modulating the glucocorticoid receptor (GR) and ERK1/2 expression [98]. The findings were further supported by an in vitro study, which showed that melatonin could reduce the DEX-induced decline in Ki67 and Nestin expression in the neurosphere of adult hippocampal NSCs by regulating the ERK1/2, cyclin E, and CDK2 via the melatonin and glucocorticoid receptors [107] (Table 10 and Table 11).

In addition, melatonin also protected against the behavioral impairments caused by morphine sulfate and ketamine in rats and mice [99,100]. It was found that the protective effects were brought by the upregulation of BDNF and modulation of the MEK/ERK and PI3K/AKT signaling pathways [100]. However, the roles of melatonin on the neurogenesis regulation in the morphine sulfate- and ketamine-exposed animals have not yet been sufficiently studied (Table 10).

Environmental toxicants such as insecticide and industrial chemicals affect neurodevelopment. Exposure of fenvalerate (FEN) 5 h after fertilization caused abnormal swimming behavior in zebrafish [101]. However, melatonin could prevent the FEN-induced abnormal behavior and neurogenesis disruption by suppressing the pro-apoptotic genes such as *Bax, Fas, caspase 8, caspase 9, and caspase 3* and upregulating the anti-apoptotic genes such as *Bcl-2* [101]. Tri-ortho-cresyl phosphate (TOCP) is a material that is widely used in industries. Melatonin protected the mouse E12.5 NSCs from TOCP-induced cell death by suppressing the ROS levels and activating the ERK1/2 signaling pathway [108] (Table 10 and Table 11).

Radiation and electromagnetic fields also affected spatial memory and cognitive functions in the rats and mice [102,103,104,105,106]. Melatonin or its metabolite *N*1-acetyl-*N*2-formyl-5-methoxykynuramine (AFMK) reduced the oxidative stress levels, improved neurogenesis, and further enhanced the behavioral outcomes of the animals after the exposure of radiation and electromagnetic fields [102,103,104,105,106] (Table 10).

Collectively, the findings suggest that melatonin exerts protective effects on the environmental factor-induced neurological deficits by modulating neurogenesis.

## 9. Melatonin and Other Diseases That Are Related to Neurogenesis Impairment

Melatonin has been found to also attenuate neurogenesis impairment in a wide range of other diseases that were not discussed above.

As a metabolic disease, diabetes mellitus (DM) can lead to neurological problems chronically. A high fat diet (HFD) and streptozotocin (STZ)-induced DM led to neurogenesis and synaptogenesis deficits and impaired spatial memory in rats [109]. Melatonin, however, could reverse the adverse effects caused DM by activating the p-ERK signaling pathway and upregulating the melatonin receptor as well as insulin receptor [109] (Table 12).

DM also causes fetal neurodevelopmental impairment. When female mice were subjected to STZ before pregnancy for 3 consecutive days and then fertilized with healthy male mice, the E11.5 and E17.5 mouse embryos showed decreased proliferation and increased premature differentiation of NSCs in the brain [110]. Nevertheless, melatonin could attenuate the STZ-induced neurodevelopmental deficits by inhibiting autophagy and preventing apoptosis in the embryonic brain [110,111]. In the in vitro experiments, it was also reported that melatonin could protect the embryonic NSCs from cell death and impairments in proliferation and differentiation in the hyperglycemia condition [110,111] (Table 12 and Table 13).

Ts65Dn (TS) mice are a commonly used model of DS, which were found to exhibit different DS phenotypes including cognitive deficits and impairments in hippocampal functions [112]. It was reported that melatonin rescued the neurogenesis suppression in the Ts65Dn (TS) mice by increasing the density and activity of glutamatergic synapses [112]. Nevertheless, in another study, it was shown that melatonin had no effect on the improvement of neurogenesis and behavioral outcomes in TS mice, but only regulated the DS-induced oxidative stress [113] (Table 12).

Seizures impair neurogenesis. Induction of seizures to a rat mother led to early embryonic neurogenesis and delayed maturation of newborn neurons in the cerebellum and the Cornus ammonis 1 (CA1) region of the hippocampus in PND0 offspring [114,115]. Olfactory NSCs isolated from schizophrenia (SCZ) patients also exhibited an abnormal increase in the potassium-evoked secretion when compared with the NSCs of the healthy control [116]. Interestingly, melatonin treatment could reverse all the abnormalities caused by seizures [114,115,116] (Table 12 and Table 13).

## 10. Melatonin and Peripheral Nerve Impairment

Apart from recusing neurogenesis impairment in the CNS, melatonin also promotes nerve regeneration after peripheral nerve injury (PNI). 

Studies showed that 30-day 1 and 10 mg/kg BW melatonin treatment could promote nerve regeneration in the rats after PNI [117,118]. It was also found that melatonin improved the upper limb functional recovery and restored the number of re-innervated motor end plates on the target muscle in the rats after PNI [117]. The protection of melatonin against PNI was suggested to be brought by the suppression of CaMKII [117]. It was also suggested that the protective effects may be related to the increase of proliferation of the Schwann cells after the melatonin treatment, given that Schwann cells are crucial for axonal guidance and nerve regeneration after PNI [118]. Melatonin was shown to promote proliferation of Schwann cells by activating ERK 1/2 via the MT1 receptor [118] (Table 14).

The modulation of the nitroxidergic system plays an important role in the protective effects of melatonin on nerve injury. After rats were subjected to hypoglossal nerve transection, melatonin suppressed NADPH-d/NOS expression and preserved superoxide dismutase (SOD) activity, which increased the number of motoneurons in the hypoglossal nucleus and further improved the functional recovery [119,120]. Chronic constriction injury (CCI) is an injury model that causes peripheral neuropathic pain on the sciatic nerve in animals. One study showed that melatonin modulated the iNOS and nNOS levels in the dorsal root ganglia, which alleviated thermal nociceptive hypersensitivity in rats after CCI [121]. Oxaliplatin is a compound that is used in the treatment of colorectal cancer but it has the side effect of causing peripheral neuropathy [122]. Melatonin, moreover, was found to modulate the nitroxidergic system in oxaliplatin-treated rats. It helped reduce the oxaliplatin-induced pain behavior and neuropathic deficits by alleviating nitrosative stress [122]. Apart from regulating the nitroxidergic system, melatonin also inhibited the apoptotic pathway and activated the autophagy pathway in the sciatic nerve and dorsal root ganglia in oxaliplatin-treated rats [122] (Table 14).

Due to its antioxidant properties, melatonin promoted nerve recovery after ischemia–reperfusion (I/R) injury. Melatonin salvaged the nerve fibers from ischemic degeneration and it decreased edema and damage in the myelin sheaths and axons after the I/R injury by reversing the I/R-induced increase in the MDA levels [123]. In the cut or crush sciatic nerve injury model, melatonin also preserved myelin sheath in the injured rats by decreasing lipid peroxidation, which helped promote the recovery [124]. In both injury models, melatonin treatment increased the SOD levels [123,124] (Table 14).

Last but not least, the roles of melatonin of nerve regeneration were further confirmed by the pinealectomy experiment, in which pinealectomy was performed in the rats 3 weeks before they were subjected to a surgical intervention consisting of bilateral sciatic nerve section and primary suture repair [125]. The results showed that the collagen content of the sciatic nerve and macroscopic neuroma formation increased in pinealectomy rats, but melatonin treatment reversed the changes [125]. Moreover, melatonin also helped reduce the stimulus intensities required to excite a nerve action potential response (NAP) in the pinealectomy rats [125] (Table 14).

## 11. Conclusions

From the traditional biological perspective, melatonin is well recognized as a key player of regulating circadian rhythm. Recent studies, however, have highlighted the pleiotropic nature of melatonin, which is widely related to the normal functioning of different systems, including the CNS. Due to its diverse effects, the melatonin signaling system could be a possible treatment target of disorders of the CNS. As increasing evidence has shown that melatonin is pro-neurogenic, it is a possible drug candidate that may be used for treating neurogenesis-related disorders, such as mood disorders, neurotrauma, and neurodegenerative disorders. However, as the neurogenic and treatment effect of melatonin has been just discovered in the past two decades, its potential as a drug remains to be further confirmed and explored. 

Further exploration of the signaling mechanisms, treatment effect of the synthetic agonist/antagonist on different disorders, and potential side effects are possible future study directions in terms of applying melatonin in treating various CNS disorders. The lack of severe side effects and the affordable nature of melatonin would imply its applicability in clinical situations for chronic treatment.

## Figures and Tables

**Table 1:** Table showing the main findings regarding the effects of melatonin or its analog(s) on neurogenesis and the respective molecular mechanisms in the in vitro studies.

Cells	Lowest Dosage Causing an Observable Effect	Main Findings	Molecular Mechanisms	References
Mouse cortical NSCs (E14)	0.05 μM, 28-h treatment of melatonin	Increased NSC viability and proliferation Enhanced glial and oligodendrocyte differentiation	Not examined in the study	Ghareghani et al., 2017 [22]
Mouse cortical NSCs (E12.5)	100 nM melatonin, 3-day treatment of melatonin	Promoted neuronal differentiation	Melatonin receptor MT1; ERK signaling pathway; CBP/p300-mediated acetylation of histone H3 lysine 14	Li et al., 2017 [23]
Mouse cortical NSCs (E14)	10^−3^ µM, 48-h treatment of melatonin or its analog IQM316	Increased neuronal precursor marker expression (DCX)	Melatonin receptors (for the actions of melatonin but not the actions of IQM316)	Figueiro-Silva et al., 2018 [24]
Rat PC12 cells	10 μM, 24-h treatment of melatonin	Enhanced cell proliferation Induced neurite outgrowth Promoted neuronal differentiation	Melatonin receptors MT1/MT2; MEK/ERK and PI3K/AKT signaling pathways	Liu et al., 2016 [25]
Mouse C17.2 cells	0.05 nM, 24-h treatment	Increased neuronal differentiation	Melatonin receptor MT1; Histone H3 acetylation	Sharma et al., 2008 [26]
Rat midbrain NSCs (E14)	1 nM, 3-day treatment of melatonin	Increased NSC viability Promoted dopaminergic neuronal differentiation and decreased astrocyte production	Melatonin receptors MT1/MT2; BDNF and GDNF	Kong et al., 2008 [27]
Mouse NSCs (E15.5; from ganglionic eminence)	5 μM, 4-day treatment of melatonin	Suppressed epidermal growth factor (EGF)-stimulated NSC proliferation Treatment during the proliferation period promoted 1% FBS-induced neural differentiation of NSCs Treatment during the differentiation period decreased neural differentiation of the NSCs	Not examined in the study	Moriya et al., 2007 [28]
Human amniotic fluid mesenchymal stem cells (16 to 20 week of pregnancy)	0.1 μM, 2-day treatment of melatonin	Increased levels of dopaminergic neuron markers (TH and NURR1) Increased levels of neuronal protein marker (βIII-tubulin) Decreased levels of glial fibrillary acidic protein markers (GFAP)	ERK phosphorylation; CaMKII signaling pathway	Phonchai et al., 2019 [29]
Mouse-induced pluripotent stem cells	1 μM, 7-day treatment of melatonin	Improved the differentiation of iPSCs into NSCs Promoted the differentiation of iPSC-derived NSCs into neurons	Melatonin receptors MT1/MT2; PI3K/AKT signaling pathway	Shu et al., 2016 [30]
52–56-day-old adult rat hippocampal organotypic culture	10^−11^ M, 6-h treatment of melatonin	Stimulated dendrite growth and dendrite formation Increased dendrite complexity	Not examined in the study	Domínguez-Alonso et al., 2012 [31]
52–56-day-old adult rat hippocampal organotypic culture	100 nM, 6-h treatment of melatonin	Stimulated dendritogenesis	Melatonin receptors MT1/MT2; CaMKII and PKC ERK1/2	Domínguez-Alonso et al., 2015 [32]
Mouse adult hippocampal NSCs	10^−8^ μM, 48-h treatment of melatonin	Increased neuronal differentiation Promoted NSC survival	Melatonin receptors MT1/MT2	Ramírez-Rodríguez et al., 2009 [33]
Mouse adult subventricular zone NSCs (8-week-old mice)	0.001 μM, 7-day treatment of melatonin	Increased NSC proliferation Promoted neuronal differentiation	Melatonin receptor MT1	Sotthibundhu et al., 2010 [34]
Mouse adult subventricular zone NSCs (8-week-old mice)	100 µM, 7-day treatment of melatonin	Increased NSC proliferation	Melatonin receptors MT1/MT2; ERK/MAPK signaling pathway	Sotthibundhu et al., 2016 [35]
Rat adult hippocampal NSCs (2-month-old rat)	0.5 μM, 3-day treatment of melatonin	Increased NSC proliferation	Melatonin receptors MT1/MT2; phosphorylation of ERK1/2 and c-Raf	Tocharus et al., 2014 [36]
Mouse adult spinal cord stem/progenitor cells (6-week-old mice)	0.01 μM, 7-day treatment of melatonin	Promoted proliferation and maintenance of NSCs	Melatonin receptors MT1/MT2; PI3K/AKT signaling pathway	Yu et al., 2019 [37]

**Table 2:** Table showing the main findings regarding the effects of melatonin or its analog(s)/receptor antagonist on neurogenesis and the respective molecular mechanisms in the in vivo studies.

Animals	Treatment Timeline and Dosage	Brain Regions	Main Findings	Behavioral Outcomes	Molecular Mechanisms	References
3-month-old male C57BL/6j mice	2 mg/kg BW melatonin or its analog IQM316, administered daily for 7 days or 28 days	Dentate gyrus	Melatonin and IQM316 increased BrdU+/NeuN+ cells	IQM316 improved memory	Modulation of mitochondrial DNA copy number and oxidative phosphorylation proteins (COX I, COX IV, ATP-5β, and NDUFB8)	Figueiro-Silva et al., 2018 [24]
Adult C57BL/6 mice	8 mg/kg BW melatonin, intraperitoneally injected daily for 14 days	Dentate gyrus	Increased number of DCX+ neuronal precursor cells Promoted dendritic maturation of immature neurons	Not examined in the study	Not examined in the study	Ramirez-Rodriguez et al., 2011 [38]
8-week-old male BALB/c mice	≈8 mg/kg BW melatonin, via drinking water for 1 or 6 months	Dentate gyrus	Modulated structural plasticity of mossy fiber projection	N/A	Not examined in the study	Ramírez-Rodríguez et al., 2018 [39]
1-month-old male SD rats	Rats were pinealectomized and treated with 6mg/L melatonin in drinking water starting from 1 week after surgery for 2, 4, 6, 8, 10, or 17 months	Dentate gyrus	Promoted neurogenesis in the pinealectomized rats	Not examined in the study	Not examined in the study	Rennie et al., 2009 [40]
Pregnant rats’ offspring (PND35-77)	8-week-old female pregnant rats subjected to pinealectomy and allowed to mate with adult males 30 days after surgery	Subgranular zone	Male offspring with no melatonin treatment showed lower number of Ki-67+-proliferative cells when compared to the offspring which received melatonin treatment during gestation and lactation period	Male offspring showed deficits in righting reflex, free-fall righting and walking Spatial reference and working memory were disrupted But melatonin alleviated the behavioral deficits	Not examined in the study	Motta-Teixeira et al., 2018 [41]
Female 8-week-old C57BL/6 mice	8 mg/kg BW melatonin, intraperitoneally injected daily for 7 or 14 days	Dentate gyrus	Increased survival of new-born neurons and increased the number of intermediate neuronal progenitor cells and immature post-mitotic neurons	Reduced antidepressant-like behavior (Porsolt forced swim test)	Not examined in the study	Ramírez-Rodríguez et al., 2009 [33]
Adult male BALB/c mice	8 mg/kg BW melatonin, orally administered daily for 3, 6, 9, or 12 months	Dentate gyrus	Increased proliferation and survival of new cells and increased number of DCX+ cells	Not examined in the study	Not examined in the study	Ramírez-Rodríguez et al., 2012 [42]
8-week-old male BALB/c mice	8 mg/kg BW melatonin, orally administered every 3 days for 1, 3, or 6 months	Dentate gyrus	Increased number of calretinin+ neurons	Not examined in the study	Not examined in the study	Ramírez-Rodríguez, et al., 2014 [43]
2–3-month-old male C3H/HeN mice	0.02 mg/mL melatonin in drinking water for 12 days	Dentate gyrus	Potentiated running-wheel activity-induced cell survival and neurogenesis	Not examined in the study	Not examined in the study	Liu, et al., 2013 [44]
Female C57BL/6 mice	10 mg/kg BW luzindole, administered daily for 14 days	Subgranular zone	Decreased DCX+ neuronal precursor cells and decreased Ki-67+ proliferative-cells; increased GFAP+/Sox2+ neural stem cells	No effect on the hopeless behavior	Melatonin receptors MT1/MT2	Ortiz-López et al., 2016 [45]

**Table 3:** Table showing the main findings regarding the effects of melatonin and its receptor agonist on neurogenesis in depression and the respective molecular mechanisms in the in vivo studies.

Animals	Treatment Timeline and Dosage	Brain Regions	Main Findings	Behavioral Outcomes	Molecular Mechanisms	References
Male BALB/c mice	0.5, 1.0, 2.5, 5.0, and 10 mg/kg BW melatonin, intraperitoneally injected once daily for 14 days	Dentate gyrus	Increased number of DCX+ neuronal precursor cells Modulated dendrite maturation and complexity of new-born neurons	Decreased depression-like behavior (forced swim test)	Not examined in the study	Ramírez-Rodríguez et al., 2020 [50]
7–8-week-old male CD1 mice subjected to corticosterone for 7 weeks	10 mg/kg BW melatonin, intraperitoneally injected for 3 weeks	Dentate gyrus	Prevented corticosterone-induced reduction in cell proliferation	Reduced depression- and anxiety-like behavior (forced swim test, open field test, and novelty suppressed feeding test)	Not examined in the study	Crupi et al., 2010 [51]
6-week-old mice with C57BL/6H background subjected to corticosterone via drinking water for 14 days	10 mg/kg BW melatonin, intraperitoneally injected twice daily for 12 days	Hippocampus	Increased neurogenesis	Improvement in depression- and anxiety-like behavior (dark–light box and novelty suppressed feeding tests)	Acid sphingomyelinase/ceramide system	Hoehn et al., 2016 [52]
11-week-old male Wistar rat subjected to chronic unpredictable mild stress for 28 days	10 mg/kg BW melatonin, intraperitoneally injected daily for 4 weeks	Hippocampus	Effects on neurogenesis were not examined in the study	Decreased depression-like behavior (forced swim test)	Decreased VMAT2 expression and increased MAO-A expression	Stefanovic et al., 2016 [53]
8-week-old male BALB/c mice	1.28, 2.57, 5.13, and 10.26 mg/kg BW combination of melatonin plus citalopram, intraperitoneally injected once daily for 14 days	Dentate gyrus	Increased cell proliferation and survival Increased number of DCX+ neuronal precursor cells	Decreased depression-like behavior (forced swim test)	Not examined in the study	Ramírez-Rodríguez et al., 2014 [54]
Female SD rats subjected to prenatal restraint stress (starting from E11 until giving birth) (Bright light (650 Lux))	2–3-month-old male offspring were treated with 10–50 mg/kg BW agomelatine, intraperitoneally injected once daily for 3 or 6 weeks	Dentate gyrus	Increased survival of the proliferative cells Increased neurogenesis	Reduced depression- and anxiety-like behavior (forced swim test and elevated plus maze)	Increased p-CREB, mGlu2/3, and mGlu5 receptor levels	Morley-Fletcher et al., 2011 [55]
12-week-old female Wistar rats subjected to light stress for 1 week	10 and 40 mg/kg BW agomelatine, intraperitoneally injected once daily for 15 days	Hippocampus	Increased neurogenesis	Not examined in the study	BDNF; apoptotic signaling pathway (Bcl-2 and caspase 3)	Yucel et al., 2016 [56]
8-week-old male SD rats	40 mg/kg BW agomelatine, intraperitoneally injected once daily for 14 days	Dentate gyrus	Increased number of Ki 67+ cells No effect on cell proliferation in rats which were implanted with corticosterone pellet with addition of corticosterone injection	Not examined in the study	5HT2C receptor	AlAhmed et al., 2010 [57]
7-week-old male Wistar rats	40 mg/kg BW agomelatine, intraperitoneally injected once daily for 8, 15, or 21 days	Hippocampus	Increased neuronal maturation, cell survival, and cell proliferation	Not examined in the study	BDNF; ERK1/2, AKT, and GSK3β signaling pathways	Soumier et al., 2009 [58]

**Table 4:** Table showing the main findings regarding the effects of melatonin on neurogenesis in aging and neurodegenerative diseases and the respective molecular mechanisms in the in vivo studies.

Animals	Treatment Timeline and Dosage	Brain Regions	Main Findings	Behavioral Outcomes	Molecular Mechanisms	References
Male C57BL/6 mice subjected to d-galactose (d-gal) for 7 weeks (animal model of aging)	6 mg/mL melatonin in drinking water for 3 weeks	Dentate gyrus	Restored d-gal-induced reduction of Ki67+ proliferative cells and DCX+ neuronal precursor cells	Improved spatial memory (Morris water maze)	Increased Ser133-phosphorylated cyclic AMP response element binding protein; decreased d-gal-induced lipid peroxidation	Yoo et al., 2012 [59]
10-month-old senescence-accelerated mice (SAMP8) (animal model of aging)	≈10 mg/kg BW melatonin, via drinking water (from 1 month old to 10 months old)	Brain	Effects on neurogenesis were not examined in the study	Not examined in the study	Decreased acetylated p53 and NF-κB; increased α-secretase; decreased amyloid β (Aβ); increased Bcl-_2XL_ levels	Gutierrez-Cuesta et al., 2008 [61]
8-week-old male C57BL/6 mice treated with methamphetamine (METH) (animal model of Parkinson’s disease)	5 mg/kg BW melatonin, subcutaneously injected for 7 days	Hippocampus	Increased expressions of Nestin, DCX, and Beta-III tubulin	Not examined in the study	Attenuated the METH-induced change in MAPK signaling activity, NMDA receptor subunits (NR2A and NR2B) and CaMKII	Singhakumar et al., 2015 [62]
B6C3-Tg (APPswe, PSEN1dE9)85Dbo/Mmjax transgenic male mice (animal model of Alzheimer’s disease) or mice subjected to 10 doses of 1-methyl-4-phenyl-1,2,3,6-tetrahydropyridine (MPTP) and probenecid over 5 weeks (animal model of Parkinson’s disease)	Transplanting 25 μM melatonin-pretreated SVZ NSCs in both disease models	Hippocampus or striatum	Effects on neurogenesis were not examined in the study	Reduced Aβ plaques in the AD transgenic mouse model Improved neuronal restoration in the PD mouse model	Not examined in the study	Mendivil-Perez et al., 2017 [63]
Adult male SD rats subjected to 6-hydroxydopamine (6 OHDA) in the striatum by stereotaxic injection (animal model of Parkinson’s disease)	4.0 μg/mL melatonin in drinking water for 39 days or 4.0 μg/mL melatonin in drinking water for 39 days, plus C17.2 NSC transplantation	Striatum and substantia nigra	Combination treatments preserved tyrosine hydroxylase (TH) immunoreactivity	Reduced apomorphine-induced rotations	Not examined in the study	Sharma et al., 2007 [64]
8-week-old male C57BL/6 mice given 0.2% cuprizone in the chow diets (animal model of demyelinating disease)	6 mg/L melatonin in drinking water for 6 weeks	Dentate gyrus	Ameliorated cuprizone-induced reduction of DCX+ neuronal precursor cells and Ki-67 proliferating cells	Not examined in the study	Increased glucose utilization (GLUT3); increased pCREB; increased BDNF	Kim et al., 2019 [65]
APP/PS1 mice (animal model of Alzheimer’s disease)	≈0.1 mg/kg BW melatonin, via drinking water (starting from 4 to 8 months of age)	Cortex and the hippocampus	Effects on neurogenesis were not examined in the study	Reduced spatial learning and memory deficits	Increased levels of mitochondrial biogenesis factors; enhanced mitochondrial DNA copy number; reduced Aβ	Song et al., 2018 [66]

**Table 5:** Table showing the main findings regarding the effects of melatonin on neurogenesis in neurodegenerative disease(s) and the respective molecular mechanisms in the in vitro studies.

Cells	Lowest Dosage Causing an Observable Effect	Main Findings	Molecular Mechanisms	References
Rat adult hippocampal NSCs treated with 500 μM METH for 2 days (Parkinson’s disease)	1 μM, 2-day treatment of melatonin (starting from 30 min prior to the METH treatment)	Reversed METH-induced inhibition of NSC proliferation	Reversed METH-induced changes in tumor suppressor p53, cycle inhibitor p21CIP, NMDA receptor subunits (NR2A and NR2B), and CaMKII	Ekthuwapranee et al., 2015 [68]

**Table 6:** Table showing the main findings regarding the effects of melatonin on CNS injuries and the respective molecular mechanisms in the in vivo studies.

Animals	Treatment Timeline and Dosage	Brain Regions	Main Findings	Behavioral Outcomes	Molecular Mechanisms	References
Male ICR mice subjected to middle cerebral ischemic/reperfusion injury (CI/R)	5 or 10 mg/kg melatonin, intraperitoneally injected once daily (starting from 2 h after CI/R) or 10 mg/kg melatonin, 30 min before CI/R, and then intraperitoneally injected daily	Cortex	Melatonin increased DCX+ and ki67+ cells The ki67+ cells were Nestin+ and NG2+ and expressed neurodevelopmental proteins (adam11 and adamts20)	Improved survival Reduced cerebral infarction Improved neural functions	Melatonin receptors; preserved blood–brain barrier (BBB) integrity; reduced free radical production; decreased gp91phox cell infiltration	Chern et al., 2012 [69]
Male SD rats subjected to middle cerebral artery occlusion (MCAO) (focal cerebral ischemia)	5 mg/kg BW melatonin, intraperitoneally injected 30 min before and after ischemia/reperfusion (I/R)	Subventricular zone and white matter areas	Melatonin increased (PCNA+NG2) + oligodendrocyte progenitor cells after MCAO	Reduction in infarct volume and white matter damage	TLR4, NF-κB, and IL-1β	Zhao et al., 2019 [70]
Adult male C57Bl6/j mice subjected to mild focal cerebral ischemia	4 mg/kg BW melatonin, single i.p. bolus injection at 24 h after reperfusion, plus 0.025 mg/mL melatonin in drinking water (starting from 24 h after reperfusion and continued for 29 days)	Striatum, cortex, and lateral ventricle	Melatonin increased neuronal survival and enhanced neurogenesis	Improvement in motor and coordination deficits (grip strength and rotarod tests) Attenuated hyperactivity and anxiety (open field test)	Not examined in the study	Kilic et al., 2008 [71]
3- to 4-month-old Mongolian gerbils subjected to global forebrain ischemia	10 mg/kg BW melatonin, intraperitoneally injected 30 min prior ischemia followed by injections at 1, 2, and 6 h after occlusion (acute treatment) or 0.6mg/kg BW melatonin daily, via drinking water (starting from 2 weeks before ischemia until the end of experiment) (chronic treatment)	Dentate gyrus and CA1 hippocampus	DCX+ neuronal precursor cells increased after ischemia, and acute and chronic melatonin treatment reduced the number of DCX+ cells	Chronic but not acute melatonin attenuated ischemia-induced hyperactivity 3 days after occlusion (open field test)	Not examined in the study	Rennie et al., 2008 [72]
Adult male SD rats subjected to MCAO	Mesenchymal stem cells (MSCs) were pretreated with 5 mM melatonin for 24 h and were transplanted into the striatum of the ipsilateral hemisphere	Subventricular zone, striatum, and cortex	Melatonin increased angiogenesis and neurogenesis after MCAO	Improved behavioral outcomes (neurological scores, rotarod test, and elevated body swing test)	Increased VEGF; improved MSC survival via ERK1/2 signaling pathway through melatonin receptors	Tang et al., 2014 [73]
8-week-old SD rats subjected to spinal cord injury (SCI)	10 mg/kg BW melatonin, subcutaneously injected twice daily (starting from 1 day after SCI until the end of experiment) or melatonin treatment plus treadmill exercise	Peri-lesion site	Combination treatment increased numbers of BrdU+ proliferative cells and Nestin+ NSCs after SCI	Combination treatment improved hindlimb function Melatonin treatment alone or combination treatment increased dendritic spine density	Not examined in the study	Lee et al., 2014 [74]
Postnatal day (PND1) mice subjected to hypoxia (5% oxygen and 95% nitrogen) for 2 h	10 mg/kg BW melatonin, intraperitoneally injected 1 h before hypoxia and then daily for 3 days	Hippocampus	Melatonin increased BrdU+ proliferating cells and DCX+ neuronal precursor cells after hypoxia Reduced cell death	Attenuated neurobehavioral deficits (sensorimotor performance, locomotor functions, and hyperactivity) Improved learning and memory performance (Morris water test)	Reduced microglial activation; reduced TNFα, interleukin-1β, and nitric oxide; inhibited NF-κB activation	Wang et al., 2013 [75]
PND7 SD rats subjected to kainic acid (KA)-induced neurodevelopmental injury	10 mg/kg BW melatonin, intraperitoneally injected daily for 6 days (starting at 4 h after KA administration)	Hippocampus	Melatonin prevented hippocampal neuronal loss but had no effect on neurogenesis	Not examined in the study	Not examined in the study	Csernansky et al., 2006 [76]

**Table 7:** Table showing the main findings regarding the effects of melatonin on neurogenesis in CNS injuries and the respective molecular mechanisms in the in vitro studies.

Cells	Lowest Dosage Causing an Observable Effect	Main Findings	Molecular Mechanisms	References
Mouse cortical NSCs (E12.5) subjected to hypoxia (95% N_2_ and 5% CO_2_) for 12 h	100 nM melatonin, treated before hypoxia	Melatonin increased proliferation and neuronal differentiation of NSCs during hypoxia	MT1 receptor; phosphorylation of ERK1/2	Fu et al., 2011 [77]
Rat NSCs (E13.5) transfected with miR-363 (vitamin A deficiency (VAD)-induced congenital spinal deformities model)	1 ng/mL melatonin, treated for 24 h	Melatonin promoted proliferation, increased Nestin expression, and promoted neuronal differentiation in miR-363-transfected NSCs	Notch signaling	Li et al., 2019 [78]

**Table 8:** Table showing the main findings regarding the effects of melatonin or its precursor on neurogenesis in sleep deprivation and “jet lag” and the respective molecular mechanisms in the in vivo studies.

Animals	Treatment Timeline and Dosage	Brain Regions	Main Findings	Behavioral Outcomes	Molecular Mechanisms	References
PND 60 male BALB/c mice subjected to sleep deprivation (SD) for 96 h	10 mg/kg BW melatonin, intraperitoneally injected daily for 4 days	Dentate gyrus	Restored the reduction in the number of Sox2+/BrdU+ NSCs	Not examined in the study	Increased MECP2; reduced SIRT1; increased let-7b, mir-132, and mir-124	Hinojosa-Godinez et al., 2019 [81]
Adult male BALB/c mice subjected to SD for 96 h	10 mg/kg BW melatonin, via drinking water (for 14 days before SD, and during SD until the end of the experiment)	Subgranular zone	Increased BrdU/Nestin+ NSCs	Not examined in the study	Increased Bcl-2 and Bcl-xL	López-Armas et al., 2016 [82]
2-to 3-month-old C57BL/6 mice or C3Hf (+/+) mice subjected to SD for 96 h	20 mg/kg BW *N*-acetylserotonin (NAS) (immediate precursor of melatonin), intraperitoneally injected every 12 h during SD	Dentate gyrus	Protected SD-induced suppression of NSC proliferation	Not examined in the study	TrkB and its downstream signaling pathway	Sompol et al., 2011 [83]
6-week-old female C57Bl/6N mice subjected to 3 weeks of photoperiod alterations simulating “jet lag”	10 mg/kg BW melatonin, intraperitoneally injected daily until the end of experiment (2.5 weeks or 4 weeks)	Dentate gyrus	Prevented the reduction of cell proliferation	Prevented cognitive deficits	Not examined in the study	Iggena et al., 2017 [84]

**Table 9:** Table showing the main findings regarding the effects of melatonin on neurogenesis in inflammation and oxidative stress and the respective molecular mechanisms in the in vitro studies.

Cells	Lowest Dosage Causing an Observable Effect	Main Findings	Molecular Mechanisms	References
Mice cortical NSCs (E14) exposed to 100 ng/mL or 1 μg/mL LPS	100nM melatonin, treated before and 1 day after LPS exposure	Suppressed LPS-induced inflammation and nitric oxide (NO) production Prevented LPS-induced cell death and apoptosis Increased SOX2 expression	PI3K/Akt/Nrf2 signaling	Song et al., 2015 [91]
iPSC-derived NSCs pretreated with 500 μM H_2_O_2_	1 μM melatonin, treated for 3 days	Increased proliferation of iPSC-derived NSCs Stabilized the mitochondrial membrane potential Prevented H_2_O_2_-induced apoptosis	Melatonin receptors; PI3K/AKT signaling pathway	Shu et al., 2018 [92]
Rat NSCs (E13.5) treated with IL-18	10 ng/mL melatonin, treated for 3 days	Reduced IL-18-induced inhibition of proliferation, neurosphere formation, and neuronal differentiation	BDNF and GDNF	Li et al., 2017 [93]

**Table 10:** Table showing the main findings regarding the effects of melatonin or its precursor and metabolite on neurogenesis impairment caused by environmental factors and the respective molecular mechanisms in the in vivo studies.

Animals	Treatment Timeline and Dosage	Brain Regions	Main Findings	Behavioral Outcomes	Molecular Mechanisms	References
Male 8-week-old ICR mice subjected to scopolamine (Sco) for 2 or 4 weeks	10 mg/kg melatonin, intraperitoneally injected for 2 or 4 weeks	Dentate gyrus	Restored the decrease in DCX+ neuronal precursor cells and Ki67+ proliferative cells	Improved spatial learning and short-term memory impairment (Morris water maze test and passive avoidance test)	Not examined in the study	Chen et al., 2018 [94]
4–5-week-old male SD rats exposed to 5-fluorouracil (5-FU)	8 mg/kg BW melatonin, intraperitoneally injected for 21 or 42 days	Subgranular zone	Ameliorated the reduction in neurogenesis	Reversed spatial memory deficits	Not examined in the study	Sirichoat et al., 2020 [95]
4–5-week-old rats exposed to valproic acid (VPA)	8 mg/kg BW melatonin, intraperitoneally injected once daily for 14 days after VPA exposure or 28 days during and after VPA exposure	Subgranular zone	Prevented VPA-induced neurogenesis impairment	Prevented impairment in spatial and non-spatial memory (novel object location (NOL) test)	Not examined in the study	Aranarochana et al., 2019 [96]
4–5-week-old male SD rats exposed to methotrexate (MTX)	8 mg/kg BW melatonin, intraperitoneally injected for 15 days before and during MTX treatment or 15 days after MTX treatment, or 30 days during and after MTX treatment	Subgranular zone	Prevented MTX-induced inhibition of cell proliferation Increased cell survival rate after MTX exposure Reversed the decrease in the number of immature neurons caused by MTX	Ameliorated MTX-induced spatial memory impairment (novel object recognition (NOR) test)	Not examined in the study	Sirichoat et al., 2019 [97]
8-week-old male ICR mice exposed to dexamethasone (DEX)	8 mg/kg BW melatonin, intraperitonially injected 30 min before DEX exposure	Dentate gyrus	Restored the DEX-induced reduction in DCX and BrdU expression	Reversed DEX-induced depressive-like behavior (forced swim test)	Prevented DEX-induced reduction in glucocorticoid receptor (GR); ERK1/2	Ruksee et al., 2014 [98]
PND8 male Wistar rats exposed to morphine sulfate	50 mg/kg BW melatonin, intraperitoneally injected 30 min before the formalin test at PND30 and 60	Only effects on behavioral outcomes were measured. Effects on the brain were not examined in the study	Effects on neurogenesis were not examined in the study	Reversed the nociceptive response induced by morphine	Not examined in the study	Rozisky et al., 2016 [99]
Swiss albino mice and C57BL/6 mice strains exposed to ketamine	1 mg/kg BW melatonin or 20 mg/kg BW *N*-acetylserotonin (NAS), intraperitonially injected 30 min before ketamine exposure	Hippocampus	Effects on neurogenesis were not examined in the study	Attenuated the ketamine-induced immobility in the forced swim test (FST)	MEK-ERK and PI3K-AKT pathways; BDNF	Choudhury et al., 2016 [100]
Zebrafish exposed to fenvalerate (FEN) 5 h after fertilization	10^–9^ mol/L melatonin, treated 3 h prior to FEN exposure for 120 hpf	Whole zebrafish	Reduced oxidative stress and apoptotic responses induced by FEN Suppressed the changes in the expression of neurogenesis-related genes (*Dlx2, Shha, Ngn1, Elavl3, and Gfap*) caused by FEN	Ameliorated FEN-induced abnormality in swimming behavior	Reduced malondialdehyde levels and activities of Cu/Zn superoxide dismutase (Cu/Zn SOD), catalase, and glutathione peroxidase; suppressed pro-apoptotic genes (*Bax, Fas, caspase 8, caspase 9, and caspase 3*); increased expression of anti-apoptotic gene (*Bcl-2*)	Han et al., 2017 [101]
Male offspring of female rats which were exposed to a single dose of 1 Gy 60 Co gamma rays (Gy) during pregnancy	4 mg/kg BW melatonin, via drinking water (PND14–20), or 20 mg/L/day melatonin, via drinking water (from PND 28–55)	Hilus, granular cell layer (GCL), and CA1 region of hippocampus	Melatonin increased number of BrdU+ proliferative cells in the hilus and increased number of NeuN+ neurons in the hilus and GCL in the irradiated PND21 rats Melatonin also increased number of NeuN+ neurons in the CA1 region of the irradiated PND 56 rats	Improved spatial memory (Morris water maze)	Reduced ROS levels in irradiated PND56 rats	Pipová Kokošová et al., 2020 [102]
6–8-month-old rats exposed to a single dose of 25 Gy	100 mg/kg BW melatonin, intraperitoneally injected 60 min before radiation exposure	Subventricular zone	Prevented cell apoptosis and reduced the decrease in Nestin+ cells	Not examined in the study	Increased antioxidant enzyme activity and decreased MDA levels	Naseri et al., 2017 [103]
Male 6-week-old C57BL mice exposed to 2 Gy of whole-body Fe irradiation	10 mg/kg BW *N*1-acetyl-*N*2-formyl-5-methoxykynuramine (AFMK), intraperitoneally injected 30 min before radiation exposure	Dentate gyrus	Prevented the loss of DCX+ neuronal precursor cells and Ki-67 proliferative cells	Improved spatial memory impairment	Reduced protein oxidation (carbonyl content); reduced 4-hydroxyalkeal + malondialdehyde (MDA+HAE)	Manda et al., 2008 [104]
6-week-old male C57BL mice exposed to 6 Gy of cranial X-ray	10 mg/kg BW melatonin, intraperitoneally injected 30 min before radiation exposure	Dentate gyrus	Prevented radiation-induced reduction in DCX+ neuronal precursor cells and Ki-67+ proliferative cells	Not examined in the study	8-OHdG 4-HNE	Manda et al., 2009 [105]
Male 12-week-old Wistar albino rats exposed to a 900 MHz electromagnetic fields (EMF)	50 mg/kg BW melatonin, intraperitoneally injected daily during EMF exposure	Hippocampus and cerebellum	Prevented EMF-induced cell loss	Prevented cognitive impairment (passive avoidance test)	Reduced SOD activity	Altun et al., 2017 [106]

**Table 11:** Table showing the main findings regarding the effects of melatonin on neurogenesis impairment caused by environmental factors and the respective molecular mechanisms in the in vitro studies.

Cells	Lowest Dosage Causing an Observable Effect	Main Findings	Molecular Mechanisms	References
Adult hippocampal NSCs (from 8-week-old rats) exposed to dexamethasone (DEX)	1 μM melatonin, treated 30 min before 1 μM DEX treatment, for 5 days	Prevented DEX-induced reduction in Ki67 and Nestin expression in the neurosphere	Melatonin receptors and glucocorticoid receptor; reversed the DEX-induced changes in ERK1/2, cyclin E, and CDK2	Ekthuwapranee et al., 2015 [107]
Mouse NSCs (E12.5) exposed to tri-ortho-cresyl phosphate (TOCP)	40 μM melatonin, treated before TOCP exposure for 24 h	Prevented the decrease in cell viability after TOCP exposure Prevented TOCP-induced autophagy	Reduced production of ROS; restored ERK1/2 signaling pathway	Liu et al., 2020 [108]

**Table 12:** The main findings regarding the effects of melatonin on neurogenesis in other neurogenesis impairment-related diseases and the respective molecular mechanisms in the in vivo studies.

Animals	Treatment Timeline and Dosage	Brain Regions	Main Findings	Behavioral Outcomes	Molecular Mechanisms	References
Six-week-old male Wistar rats were fed with a high fat diet (HFD) and subjected to STZ (diabetes mellitus)	10 mg/kg BW melatonin, subcutaneously injected for 4 weeks	Hippocampus	Reversed HFD + STZ-induced neurogenesis and synaptogenesis impairment	Reversed HFD+ STZ-induced spatial memory impairment	Prevented suppression of melatonin receptor and insulin receptor; upregulated p-ERK	Wongchitrat et al., 2016 [109]
E11.5 and E17.5 embryos from 8-week-old pregnant Kunming mice subjected to streptozotocin (STZ) (diabetes mellitus)	10 mg/kg BW melatonin, intraperitoneally injected into the pregnant mice (starting from E0.5 to the end of experiment)	Cortex	Prevented STZ-induced inhibition of NSC proliferation; Decreased STZ-induced premature differentiation of NSCs	Not examined in the study	Decreased autophagy	Li et al., 2019 [110]
E11.5 embryos from 8-week-old pregnant ICR mice which were subjected to STZ (diabetes mellitus)	10 mg/kg BW melatonin, intraperitoneally injected into pregnant mice (starting from E0.5 to the end of the experiment)	Forebrain	Prevented STZ-induced reduction in proliferation of NSCs Reduced STZ-induced apoptosis	Not examined in the study	Not examined in the study	Liu et al., 2015 [111]
6–6.5-month-old Ts65Dn (TS) mice (Down syndrome)	100 mg/L melatonin, via drinking water from 6–6.5 months old to 11–12 months old	Hippocampus	Reversed neurogenesis impairment Decreased reduction in the density of hippocampal granule cells Reduced synaptic inhibition Recovered hippocampal LTP	Not examined in the study	Increased density and activity of glutamatergic synapses; decreased the levels of lipid peroxidation	Corrales et al., 2014 [112]
Ts65Dn (TS) mice (Down syndrome)	100 mg/L melatonin, treated via drinking water from the time of conception of the mouse mothers to the age of 5 months of the offspring	Hippocampus and cortex	Did not reverse the decrease in cell proliferation in the TS mice But regulated brain oxidative stress	No effects on behavioral outcomes	Modulated the activity of antioxidant enzymes (superoxide dismutase and catalase); decreased the levels of lipid peroxidation	Corrales et al., 2017 [113]
PND 0 rats from rat mothers subjected to seizures induction on E13 of the pregnancy (epilepsy)	30 μg/100 g BW melatonin, intraperitoneally injected into the rat mothers for 2 months	Cerebellum	Inhibited epilepsy-induced increase in Nestin expression	Not examined in the study	Not examined in the study	Uyanikgil et al., 2005 [114]
PND 0 rats from rat mothers subjected to seizure induction on E13 of the pregnancy and pinealectomy 1 month before seizures induction (epilepsy)	30 μg/100 g BW melatonin, subcutaneously injected into the rat mothers for 2 months (starting from the date of pinealectomy surgery)	CA1 region of the hippocampus	Inhibited pinealectomy-stimulated increase in Nestin expression	Not examined in the study	Not examined in the study	Turgut et al., 2006 [115]

**Table 13:** Table showing the main findings regarding the effects of melatonin on neurogenesis in other neurogenesis impairment-related diseases and the respective molecular mechanisms in the in vitro studies.

Cells	Lowest Dosage Causing an Observable Effect	Main Findings	Molecular Mechanisms	References
Mouse cortical NSCs (E12.5) subjected to hyperglycemia	10 nM melatonin, treated for 24 h	Promoted proliferation and self-renewal of NSCs in hyperglycemia Prevented hyperglycemia-induced premature differentiation of NSCs	Decreased autophagy; activated mTOR signaling pathway	Li et al., 2019 [110]
Mouse telencephalon NSCs (E11.5) subjected to hyperglycemia	100 nM melatonin, treated for 3 days	Prevented hyperglycemia-induced inhibition of NSC proliferation Prevented hyperglycemia-induced cell death and apoptosis	ERK signaling pathway	Liu et al., 2015 [111]
Olfactory NSCs from a 28-year-old male patient diagnosed with schizophrenia (SCZ)	10^–5^ M melatonin, treated for 12 h	Attenuated the SCZ induced-abnormal increase in potassium-evoked secretion	Not examined in the study	Cercós et al., 2017 [116]

**Table 14:** Table showing the main findings regarding the effects of melatonin on peripheral nerve impairment and the respective molecular mechanisms in the in vivo studies.

Animals	Treatment Timeline and Dosage	Targets	Main Findings	Behavioral Outcomes	Molecular Mechanisms	References
Young adult male Wistar rats subjected to PNI (ESN)	1 mg/kg BW melatonin, intraperitoneally injected daily for 1 month after ESN	Peripheral nerve tissue	Enhanced expression of GAP43 and β3-tubulin	Enhanced upper limb functional recovery Increased number of re-innervated motor end plates on the target muscle	Melatonin receptors (MT1/MT2) Suppressed CaMKII	Liu et al., 2020 [117]
Young adult male Wistar rats subjected to peripheral nerve injury (PNI), which was performed by end-to-side neurorrhaphy (ESN)	1 and 10 mg/kg BW melatonin, intraperitoneally injected daily for 30 days	Nerves on target muscle	Improved nerve regeneration	Not examined in the study	Increased proliferation of Schwann cells (MT1 and phosphorylation of ERK 1/2)	Chang et al., 2014 [118]
Adult male Wistar subjected to hypoglossal nerve transection (PNI)	5 or 100 mg/kg BW melatonin, intraperitoneally injected daily for 3, 7, 14, 21, and 30 days	Hypoglossal nucleus	Increased number of motoneurons in the hypoglossal nucleus	Not examined in the study	Suppressed NADPH-d/NOS expression	Chang et al., 2000 [119]
Adult male Wistar rats subjected to hypoglossal nerve transection (PNI)	5 or 100 mg/kg BW melatonin, intraperitoneally injected daily for 3, 7, 14, 30, or 60 days	Hypoglossal motoneurons	Preserved activities of Mn-SOD, Cu/Zn-SOD, and ChAT	Promoted functional recovery	Suppressed nNOS augmentation	Chang et al., 2008 [120]
SD rats subjected to chronic constriction injury	5 mg/kg and 10 mg/kg melatonin, intraperitoneally injected on the 14th day after surgery	Dorsal root ganglia	Modulation of the nitroxidergic system	Improvement in thermal hyperalgesia	Modulated iNOS and nNOS levels	Borsani et al., 2017 [121]
SD rats exposed to oxaliplatin (peripheral neuropathy)	3 or 10 mg/kg BW melatonin, intraperitoneally injected for 28 days	Sciatic nerve and dorsal root ganglia	Ameliorated oxidative/nitrosative stress mediated by oxaliplatin Induced autophagy and inhibited apoptosis	Alleviated oxaliplatin-induced pain behavior and neuropathic deficits (cold chemical allodynia, mechanical allodynia, and mechanical hyperalgesia)	Reduced the changes in the levels of MDA nitrite, GSH, nitrotyrosine, and MnSOD Inhibited apoptotic pathway (Bcl2, BAX, caspase 3, and cytochrome C) Autophagy pathway (LC3A/3B-I and II, beclin, Atg 3, Atg 5, and Atg 7)	Areti et al., 2017 [122]
Adult male Wistar rats subjected to ischemia–reperfusion (R/I injury)	10 mg/kg BW melatonin, injected via tail vein immediately before the reperfusion period	Sciatic nerve	Salvaged the nerve fibers from ischemic degeneration Decreased edema and damage in the myelin sheaths and axons	Not examined in the study	Reversed the I/R-induced increase in MDA levels Increased SOD levels	Sayan et al., 2004 [123]
Female Wistar rats subjected to cut or crush injury	50 mg/kg BW melatonin, intraperitoneally injected after sciatic nerve injury	Sciatic nerve	Preserved myelin sheath	Not examined in the study	Decreased lipid peroxidation Increased SOD, CAD, and GPx activity	Kaya et al., 2013 [124]
Adult male Wistar rats subjected to pinealectomy 3 weeks before surgical intervention consisting of bilateral sciatic nerve section and primary suture repair	30 mg/100 g BW melatonin, subcutaneously injected after pinealectomy	Sciatic nerve/ epineurium	Increased collagen content of the sciatic nerve and macroscopic neuroma formation after pinealectomy but the elevation was reversed by the melatonin treatment Melatonin treatment reduced stimulus intensities required to excite a NAP response after pinealectomy Melatonin treatment reduced the elevation of Type I collagen and Type III collagen in epineurium after pinealectomy	Not examined in the study	Not examined in the study	Turgut et al., 2005 [125]
